# Pseudorabies detected in hunting dogs in Alabama and Arkansas after close contact with feral swine (*Sus scrofa*)

**DOI:** 10.1186/s12917-018-1718-3

**Published:** 2018-12-06

**Authors:** Kerri Pedersen, Clinton T. Turnage, Wesson D. Gaston, Paulo Arruda, Scott A. Alls, Thomas Gidlewski

**Affiliations:** 1 U.S. Department of Agriculture, Animal and Plant Health Inspection Service, Wildlife Services, National Wildlife Research Center, 4101 LaPorte Avenue, Fort Collins, Colorado, 80521 USA; 2 U.S. Department of Agriculture, Animal and Plant Health Inspection Service, Wildlife Services, 1020 Lantrip Road, Sherwood, AR 72120 USA; 3 U.S. Department of Agriculture, Animal and Plant Health Inspection Service, Wildlife Services, 602 Duncan Drive, Auburn, AL 36849 USA; 4 Veterinary Research Institute, Audubon Manning Veterinary Clinic, 1532 S. Bell Avenue #106, Ames, Iowa, 50010 USA; 5 U.S. Department of Agriculture, Animal and Plant Health Inspection Service, Wildlife Services, 2800 Lincoln Boulevard, Oklahoma City, OK 73105 USA

**Keywords:** Aujeszky’s disease, Dogs, Feral swine, Hunting, Pseudorabies, *Sus scrofa*, Wild pig

## Abstract

**Background:**

Pigs (*Sus scrofa*) are the natural hosts of pseudorabies virus (PRV), also known as Aujeszky’s disease. Infection in mammals, with the exception of humans, typically causes extreme itching, facial swelling, and excessive salivation, followed by death in non-suid species. The risk to susceptible mammals was assumed to decrease when PRV was eliminated from U.S. commercial swine in 2004, though the virus remains endemic in feral swine. Infected feral swine pose a threat to the disease-free status of the commercial swine industry, and to other animals, including dogs, that come in direct or indirect contact with them. Since dogs are commonly used for hunting feral swine, they are at high risk of exposure.

**Case presentation:**

The following report describes the progression of pseudorabies infection in dogs in two states after exposure to feral swine. The first case occurred in a dog in Alabama after participation in a competitive wild hog rodeo. The second case occurred in multiple dogs in Arkansas after hunting feral swine, and subsequent consumption of the offal. The antibody prevalence of feral swine in the two states where the dogs were exposed is also examined.

**Conclusions:**

Dogs that are used for hunting feral swine are at high risk of exposure to pseudorabies because the disease is considered endemic in feral swine in the U.S.

## Background

Pseudorabies (PRV), also referred to as Aujeszky’s disease, is a viral disease caused by Suid alphaherpesvirus 1 [1]. Though swine (i.e., suids) are the only known reservoirs or natural hosts of the disease, numerous mammals, with the exception of humans, are susceptible to infection [2]. Adult swine usually recover after infection, but high mortality rates in piglets, and abortions in pregnant sows are typical [3]. The virus can also establish latency in swine with reactivation occurring after natural stimuli, or as a result of stressors [2].

Feral swine (*Sus scrofa*) are an invasive species in the U.S. with a widespread geographic range extending across the country [4]. Although PRV was eradicated from U.S. commercial swine in 2004, the disease is still endemic in feral swine, thus posing a threat to the industry’s disease-free status [5], and potentially causing substantial economic losses [6]. Prior to elimination of the disease in commercial swine, infection in dogs (*Canis lupus familaris*) was commonly reported [7–9]. Even though PRV is prevalent in feral swine, most dog owners, veterinarians, hunters, and wildlife biologists are unaware of the risk. Dogs become infected after direct or indirect contact with infected swine; infected dogs do not shed enough virus to transmit to other dogs [10]. Ingestion is the most common route, but transmission can also occur via inhalation or minor wounds [1]. The disease is suspected when relentless itching (pruritus), excessive salivation, and sudden death are observed, especially when exposure to feral swine has occurred [7].

In some parts of the U.S., dogs are used for hunting feral swine. This typically involves dogs that are used to bay (e.g., Catahoula, Black Mouth Cur, Rhodesian ridgeback) and then dogs used to catch (e.g., Pit bull, American bulldog) the pig. However, some hunters only use bay dogs. The bay dog tracks, follows, and locates a feral swine, whereas the catch dog holds the animal with its jaws until the hunter dispatches it. Depending on the temperament of the dog and the size of the feral swine, the bay dog and the catch dog often come in direct contact with the pig, especially once the catch dog seizes the feral swine. As such, it is not surprising that dogs can be exposed to various pathogens carried by feral swine, including PRV [11].

Though PRV infection has been described in hunting dogs previously, these reports are limited. In the U.S., infection was described in three dogs after hunting feral swine in southern Oklahoma [12]. Two specific cases were reported in Florida in hunting dogs after direct contact with feral swine, and at least six other dogs displayed signs of infection after hunting feral swine in the same area of Florida [13]. In addition, confirmed cases of PRV in dogs after direct contact with wild boar have been reported in various countries including Belgium [14], Italy [15, 16], France [17], and Germany [18]. Our objective was to report the disease progression of PRV in hunting dogs in two U.S. states after close contact with feral swine. We also examined the antibody prevalence of PRV in feral swine in those same states in order to further elucidate the potential risk to dogs.

## Case presentation

### Alabama

A 6-year old intact male Plott hound dog was presented to a veterinarian on September 18, 2014 with severe self-induced facial trauma including unilateral periocular swelling, and intense pruritus. The dog had participated in a benefit wild hog rodeo in Wilcox County, Alabama from September 11–13, 2014. During the course of the three-day spectator event that included dog, trapping, and stalk hunting categories, the dog was involved in the capture and removal of 13 feral swine. On September 19, 2014, the dog had further self-induced trauma, intense pruritus, erythema, and vomited blood. The animal was vocalizing and self-mutilation of the facial region resulted in severe and diffuse lacerations and bleeding. The attending veterinarian administered morphine, but the clinical presentation including facial self-mutilation remained unaltered. By the next day (September 20th- Day 9 or 10), the dog was dead.

Fresh and 10% formalin-fixed sections of cerebrum, cerebellum, brainstem, liver, spleen and tonsil from the dog were submitted to Iowa State University Veterinary Diagnostic Laboratory (ISUVDL) in Ames, Iowa for testing. Histopathologic examination was performed on all formalin-fixed tissues. Fresh tissue sections of cerebrum, cerebellum, and brainstem were submitted for real-time polymerase chain reaction (PCR) testing for PRV, and virus isolation [19, 20].

**Fig. 1:**
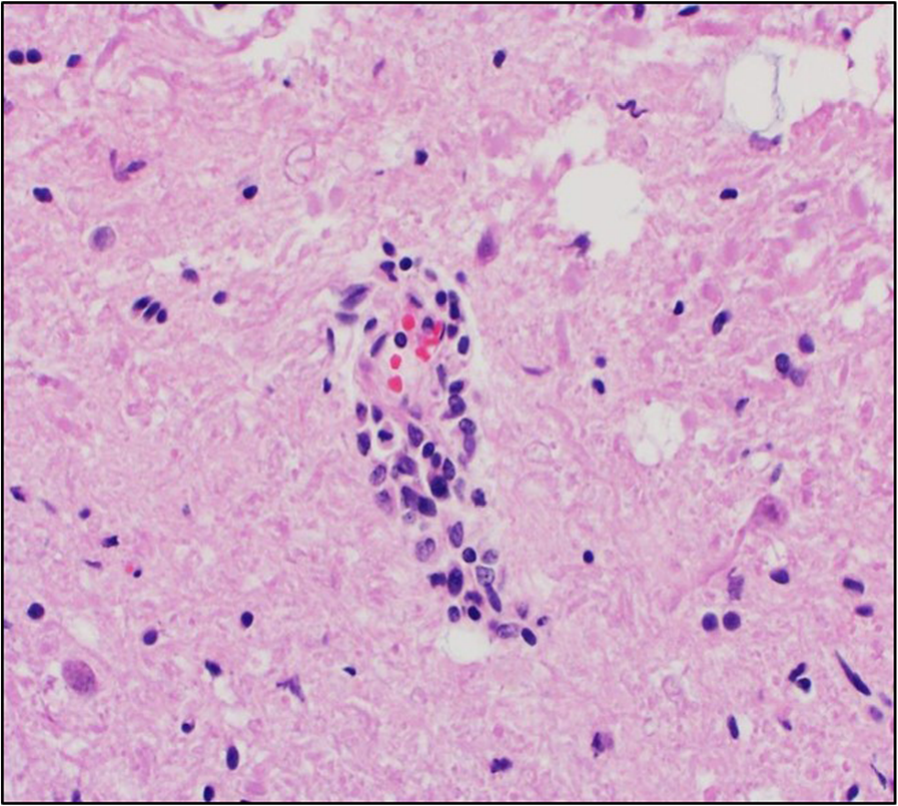
Virchow-Robin spaces expanded by low to moderate numbers of lymphocytes, plasma cells, and rare macrophages. The sample was collected from the brain tissue of a hunting dog in Alabama that developed symptoms compatible with pseudorabies virus after interacting with feral swine

**Fig. 2:**
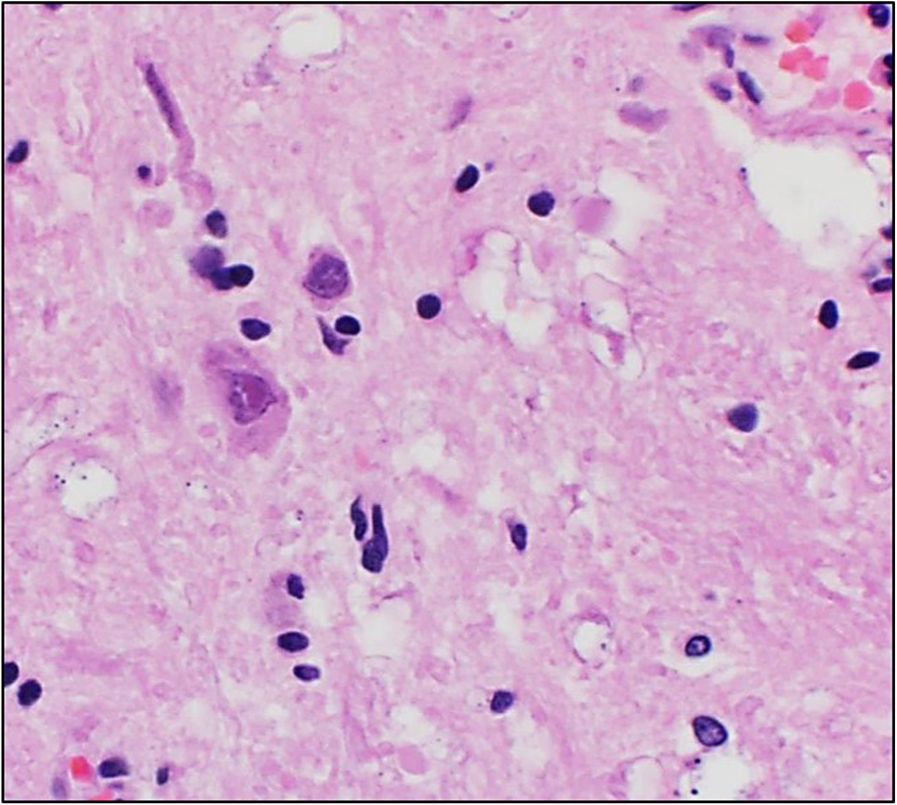
Cerebrum, neuron with an intranuclear eosinophilic inclusion and peripheralized chromatin. The sample was collected from the brain tissue of a hunting dog in Alabama that developed symptoms compatible with pseudorabies virus after interacting with feral swine

**Fig. 3:**
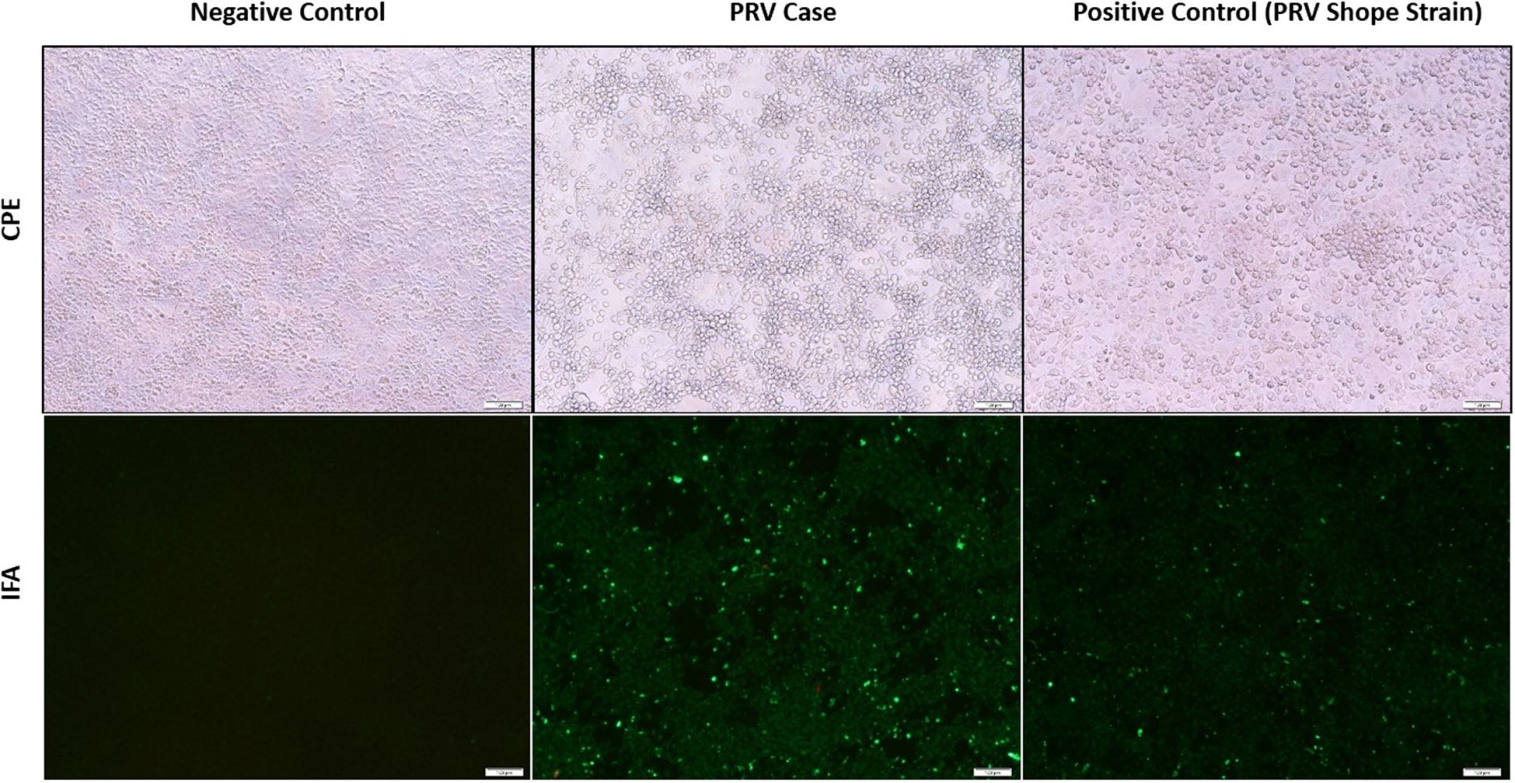
Cytopathogenic effect (CPE) and immunofluorescence antibody test (IFA) of brain tissue from a dog infected with pseudorabies virus (PRV) in Alabama

A direct fluorescent antibody test was also conducted on brain tissue for detection of rabies antigen [21]. Though no antigen was detected, rabies testing was considered inconclusive because the cerebellum and brainstem, which are the preferred tissues for rabies testing, were unavailable because they had been used for PRV testing. Histopathologic examination of the liver and spleen did not reveal any significant lesions, but moderate lymphoplasmacytic encephalitis was detected in the brainstem. Virchow-Robin spaces were often infiltrated and expanded by low to moderate numbers of lymphocytes, plasma cells, and rare macrophages (Fig. 1); multifocal areas of gliosis were also observed. Endothelial cells within affected vessels were moderately enlarged (hypertrophied). Occasionally neurons and astrocytes contained an intranuclear eosinophilic inclusion that often peripheralized the chromatin (Fig. 2). Tonsil was negative but brain tissue tested positive (cycle threshold (Ct) = 30; value < 40 considered positive) for PRV by PCR. Pseudorabies virus was isolated from central nervous system tissue in porcine kidney-15 cells, and confirmed by immunofluorescence staining (Fig. 3). Based on the clinical presentation, histologic lesions, PCR results, and virus isolation, the dog was diagnosed with PRV.

### Arkansas

Two hunters (Hunters A and B) used nine mixed breed dogs (six belonging to Hunter A, and three to Hunter B) to hunt feral swine in Sevier county, Arkansas on December 13, 2014 (Day 0). According to the hunters, the six dogs owned by Hunter A caught and bit a young female feral swine multiple times on the face and body prior to the animal being dispatched. Hunter B’s three dogs did not have direct contact with the feral swine during the hunt. However, the three dogs along with the hunter’s three other dogs (six total) reportedly consumed the offal once he finished butchering the animal at his house. On December 17, 2014 (Day 4), one of Hunter A’s dogs refused to eat, scratched its head profusely, and was whining and moaning. By December 18, 2014 (Day 5), three of Hunter A’s dogs had swollen heads and appeared to have vomited prior to dying. Two more of Hunter A’s dogs had diarrhea. Two of Hunter B’s dogs were dead and one was sick. On December 19, 2014 (Day 6), Hunter A had one dog that started vomiting, and another that was salivating excessively and then died a few hours later. Hunter B’s sick dog was euthanized because its symptoms were so severe. Two more of Hunter B’s dogs died on December 20, 2014 (Day 7), and another one was sick and then died on December 21, 2014 (Day 8). One of Hunter A’s dogs (directly exposed) was sick, but recovered by December 29th (Day 16). Hunter A had one dog that was directly exposed but failed to develop clinical signs. In the end, Hunter A lost four of six dogs that were directly exposed to the feral swine, and Hunter B lost all six of his dogs that consumed the offal.

Samples from one each of the two hunter’s dogs (one male and one female) that displayed compatible signs of PRV on December 19th and 20th respectively, and the feral swine that had been hunted were initially submitted to the Arkansas Livestock and Poultry Commission Veterinary Diagnostic Laboratory (ARVDL) in Little Rock, Arkansas. Specifically, a fecal swab, nasal swab, and serum sample from the female dog as well as the carcasses of both dogs, and the head, first cervical vertebra, and some muscle tissue from the feral swine were submitted for examination. The brain (except the frontal lobe), a section of spinal cord, and skeletal muscle tissue were fixed in formalin for histopathology. The frontal lobe and muscle tissue from the feral swine, the brain stem, trigeminal nerve, and serum from the female dog, and a sagittal section of the brain and the trigeminal nerve of the male dog were forwarded to ISUVDL for real-time PCR testing, and virus isolation. Serology was conducted with the PRV gB enzyme-linked immunosorbent assay (ELISA; HerdCheck, IDEXX Laboratories, Inc. Westbrook Maine, USA) at ISUVDL.

Histopathology conducted on the feral swine tissues was consistent with PRV. Attempts to identify unequivocal viral inclusions bodies were unsuccessful, likely due to the subjective and multifocal nature of such findings. One of the dogs had encephalitic lesions that were compatible with PRV. The other dog did not have lesions, but had inflammation in the intestine and the liver. However, multifocal areas of necrosis or eosinophilic intranuclear inclusions were not observed. The PCR results for the brain tissue from both dogs (Ct =40.0, 40.8 respectively) and the feral swine (Ct = 38.7) were close to the cut-off value, and consequently were considered suspect positive. The single serum sample was antibody negative.

### Feral swine surveillance

The U.S. Department of Agriculture’s Wildlife Services (WS) collects blood from approximately 3000 feral swine annually across the U.S. for wildlife damage management purposes. A subset of these are tested for exposure to various pathogens. Due to funding, samples are collected based on a fiscal year from October 1st to September 30th of the following year. Sera are tested routinely for exposure to PRV due to the economic and regulatory concerns related to a detection in the commercial swine industry. We examined the antibody results of 1965 feral swine sera collected in Alabama (*n* = 755) and Arkansas (*n* = 1210) from 2010 to 2017 to determine PRV serologic status of feral swine in those states.

Sera were tested at the Washington Animal Disease Diagnostic Laboratory in Pullman, Washington, the Wisconsin Veterinary Diagnostic Laboratory in Madison, Wisconsin, or the Kentucky Federal Brucellosis Laboratory in Frankfort, Kentucky using the PRV gB ELISA (HerdCheck, IDEXX Laboratories, Inc. Westbrook Maine, USA). Testing was performed according to the manufacturer’s instructions.

### Map design

**Fig. 4:**
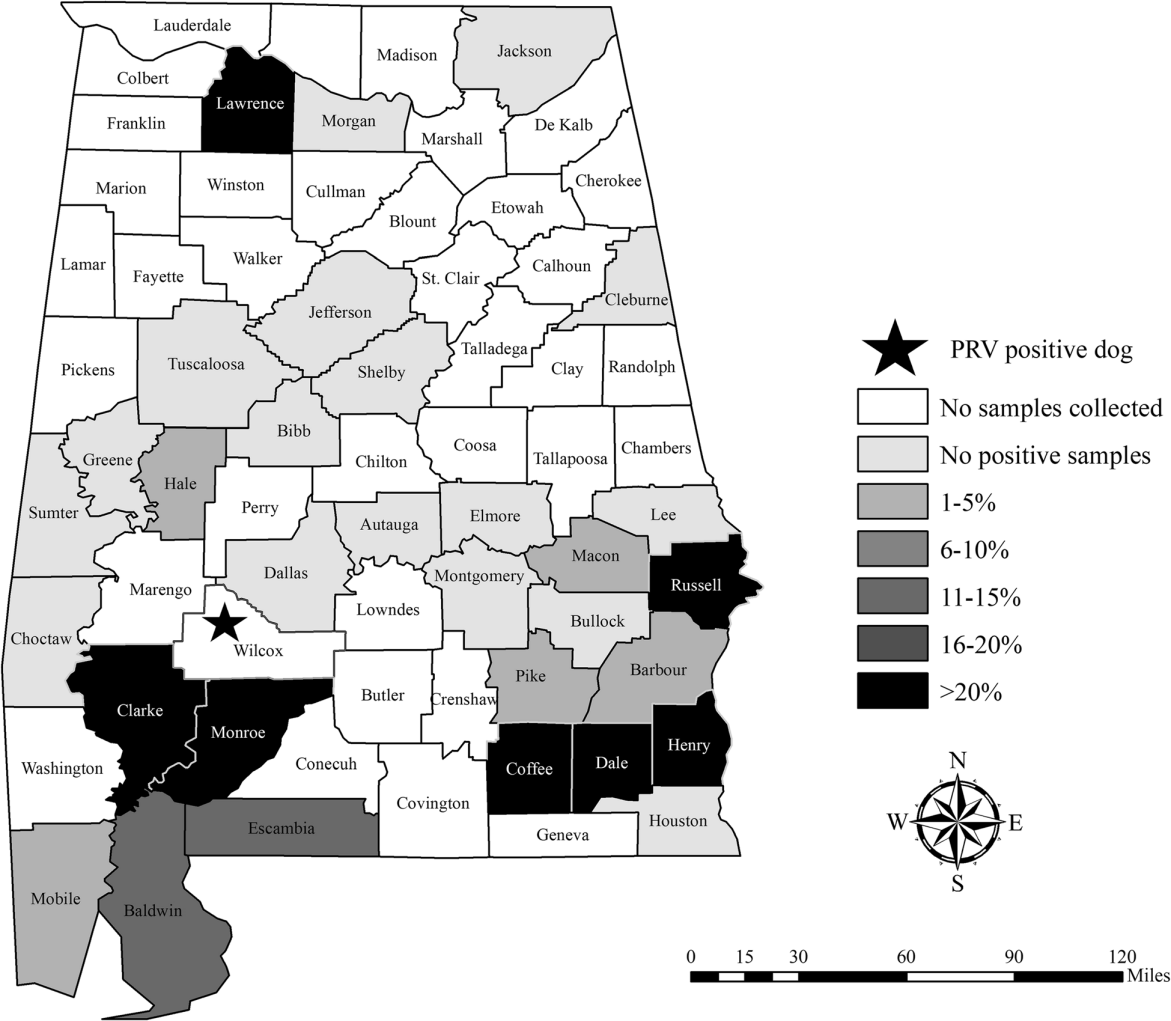
Apparent antibody prevalence of pseudorabies virus in feral swine (*Sus scrofa*) by county collected in Alabama from 2010 to 2017

**Fig. 5:**
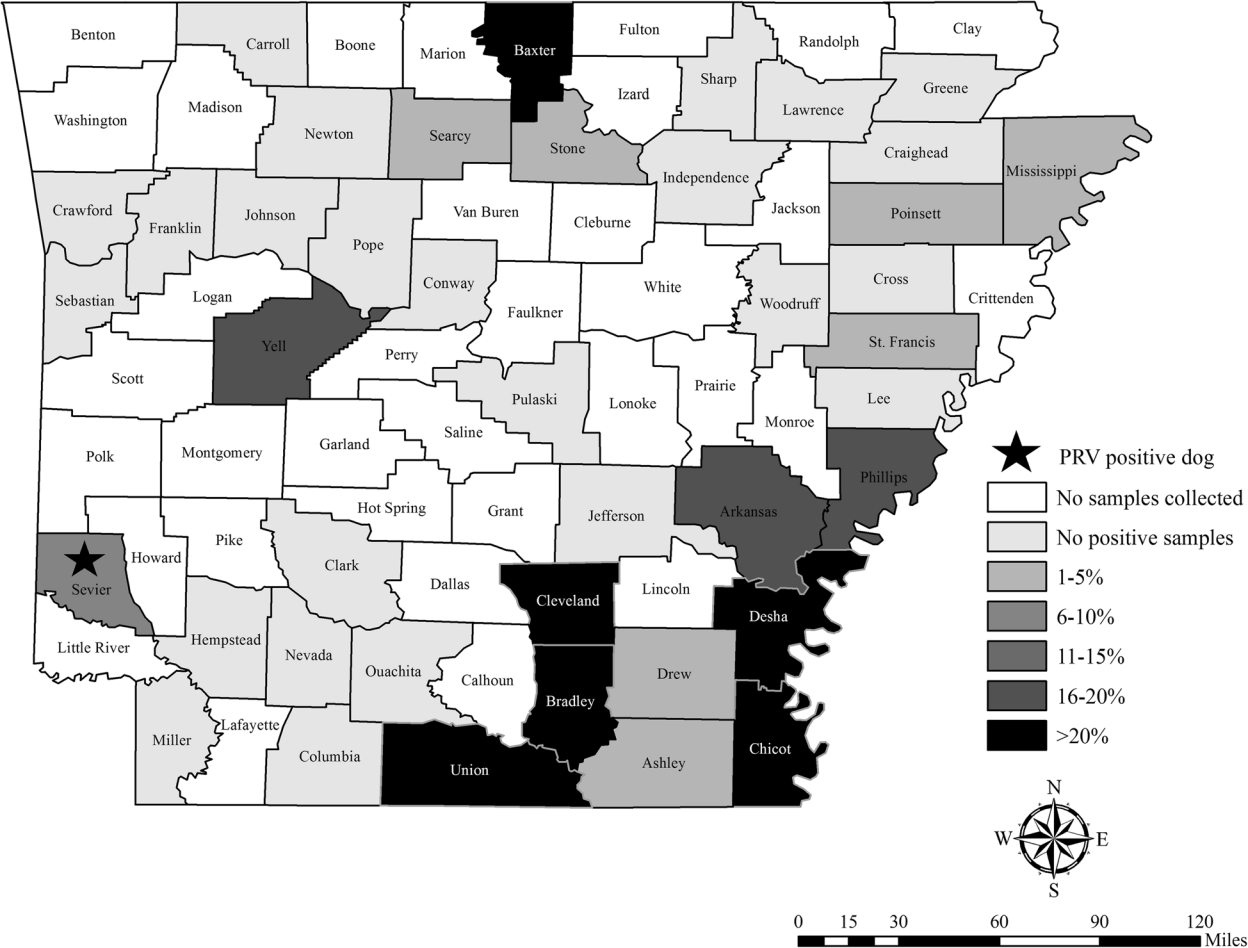
Apparent antibody prevalence of pseudorabies virus in feral swine (*Sus scrofa*) by county collected in Arkansas from 2010 to 2017

Maps of feral swine serology results were created in ArcGIS version 10.5 (ESRI, Redlands, California, USA). The PRV antibody prevalence for each county in Alabama (Fig. 4) and Arkansas (Fig. 5) was calculated, and then color coded by the following categories: no samples collected, no positive samples, 1–5%, 6–10%, 11–15%, 16–20% or > 20%. The cases described herein where dogs died from PRV after direct or indirect exposure to feral swine are marked with a star on the maps (Figs. 4 and 5).

The PRV antibody prevalence in feral swine from 2010 to 2017 in Alabama was 13.9% (95% confidence interval (CI): 11.6–16.6), and 13.1% (95% CI: 11.3–15.1) in Arkansas. There was wide-ranging variation in antibody prevalence between counties (Figs. 4 and 5) and years (Table 1).

**Table 1:** Antibody prevalence of feral swine in Alabama and Arkansas tested for exposure to pseudorabies virus from 2010 to 2017

Year	Alabama (n, % (95% CI))	Arkansas (n, % (95% CI))
2010	54, 76.6 (67.1–88.2)	150, 9.3 (5.6–15.1)
2011	100, 14.0 (8.5–22.1)	186, 11.3 (7.5–16.6)
2012	93, 8.6 (4.4–16.1)	137, 22.6 (16.4–30.3)
2013	83, 13.3 (7.5–22.2)	144, 18.1 (12.6–25.1)
2014	99, 14.1 (8.6–22.4)	175, 16.0 (11.3–22.2)
2015	81, 4.9 (1.9–12.0)	166, 7.2 (4.2–12.2)
2016	122, 23.8 (17.1–32.1)	139, 14.4 (9.5–21.2)
2017	123, 11.4 (7.4–22.3)	113, 5.3 (2.5–11.1)
All years	755, 13.9 (11.6–16.6)	1210, 13.1 (11.3–15.1)

## Discussion and conclusions

Feral swine pose a threat to the PRV-free status obtained in commercial swine in Alabama in 1998 and Arkansas in 2000 (U.S. in 2004) [22]. Though approximately 18% of U.S. feral swine are PRV antibody-positive, county-level or local prevalence varies widely [5]. We assume that the county level antibody prevalence based on this surveillance is representative even though the sample sizes are variable. Based on this assumption, in Sevier county, Arkansas, where the 10 dogs described here succumbed to PRV infection, the antibody prevalence of PRV in feral swine was 9.3% (95% CI: 5.4–15.6; *n* = 164; Fig. 5). Although this is similar to the 13.1% antibody prevalence detected across Arkansas, the antibody prevalence of feral swine was 37.5% (95% CI: 18.5–61.4) in McCurtain county, Oklahoma which borders Sevier county. Since feral swine home ranges are typically influenced by food availability and unrestricted compared to livestock, movement to a neighboring county would not be unexpected [23]. Though serological results are not equivalent to shedding rates, evidence of viral shedding has been detected in multiple tissue types suggesting various routes of transmission [24].

Two of the Arkansas hunting dogs survived presumed exposure even though mortality or euthanasia due to severe clinical symptoms occurred in all other dogs. Although one of the two dogs was identified as sick by the owner, it is unlikely that the dog was infected with PRV because mortality usually follows the development of clinical signs in dogs [25]. The dog that never developed any signs probably never actually had direct contact with the feral swine during the hunt. Both surviving dogs were not fed the offal. Even though samples were not submitted from all of the dogs, we assume that the other dogs that died were also infected with pseudorabies since they exhibited similar clinical signs.

Wild hog rodeos are a rural southern tradition in which dogs compete against wild pigs; although discontinued in most areas, the objective historically was to judge a dog based on its ability to physically restrain feral swine. Hog rodeos are now synonymous with baying competitions; feral swine are placed in a fenced area with bay dogs, and the dogs are judged based on their ability to stop the feral swine’s pursuit without touching the animal within a certain time frame (Scott Alls, personal communication). Although feral swine exist in Wilcox County, Alabama where the competition occurred, no feral swine surveillance has occurred in that county (Fig. 4). However, in Clarke and Monroe counties (bordering Wilcox), antibody prevalence in feral swine was 41.4% (95% CI: 32.2–51.3) and 50% (95% CI: 18.8–81.2) respectively. While the exact origin of the feral swine for this event is unknown, they are typically acquired locally in the same county or in nearby counties. Given the high PRV antibody prevalence in Clarke and Monroe counties, it is not surprising that some of the feral swine obtained for the event were infectious at the time of the event.

In the dogs reported here, PRV infection was assumed to have occurred after exposure to feral swine either by direct or indirect contact. Very few cases of PRV infection in dogs occurring after exposure to feral swine have been reported despite the risk. We believe that it is important for hunters, wildlife biologists, and veterinarians to be aware that the risk still exists both when hunting feral swine with dogs and/or when allowing them to consume uncooked feral swine meat, organs or carcass. If a dog should become infected, euthanasia may be the most humane option since PRV infection causes such severe symptoms in dogs and is usually fatal. Dogs that succumb to infection or are euthanized because of suspected infection should either be buried or cremated to avoid the risk of spreading the disease to other dogs.

## Abbreviations

ARVDL: Arkansas Livestock and Poultry Commission Veterinary Diagnostic Laboratory
Ct: Cycle threshold
ELISA: Enzyme-linked immunosorbent assay
gB: Glycoprotein B
ISUVDL: Iowa State University Veterinary Diagnostic Laboratory
PCR: Polymerase chain reaction
PRV: Pseudorabies virus
